# The Feasibility of Modelling the Crown Profile of *Larix olgensis* Using Unmanned Aerial Vehicle Laser Scanning Data

**DOI:** 10.3390/s20195555

**Published:** 2020-09-28

**Authors:** Ying Quan, Mingze Li, Zhen Zhen, Yuanshuo Hao, Bin Wang

**Affiliations:** Key Laboratory of Sustainable Forest Ecosystem Management-Ministry of Education, School of Forestry, Northeast Forestry University, Harbin 150040, China; quanying@nefu.edu.cn (Y.Q.); zhzhen@syr.edu (Z.Z.); haoyuanshuo@nefu.edu.cn (Y.H.); wangbin@nefu.edu.cn (B.W.)

**Keywords:** unmanned aerial vehicle (UAV), light detection and ranging (LiDAR), crown profile model, *Larix olgensis*

## Abstract

Unmanned aerial vehicle (UAV) laser scanning, as an emerging form of near-ground light detection and ranging (LiDAR) remote sensing technology, is widely used for crown structure extraction due to its flexibility, convenience, and high point density. Herein, we evaluated the feasibility of using a low-cost UAV-LiDAR system to extract the fine-scale crown profile of *Larix olgensis*. Specifically, individual trees were isolated from LiDAR point clouds and then stratified from the point clouds of segmented individual tree crowns at 0.5 m intervals to obtain the width percentiles of each layer as profile points. Four equations (the parabola, Mitscherlich, power, and modified beta equations) were then applied to model the profiles of the entire and upper crown. The results showed that a region-based hierarchical cross-section analysis algorithm can successfully delineate 77.4% of the field-measured trees in high-density (>2400 trees/ha) forest stands. The crown profile generated with the 95th width percentile was adequate when compared with the predicted value of the existing field-based crown profile model (the Pearson correlation coefficient (*ρ*) was 0.864, root mean square error (RMSE) = 0.3354 m). The modified beta equation yielded slightly better results than the other equations for crown profile fitting and explained 85.9% of the variability in the crown radius for the entire crown and 87.8% of this variability for the upper crown. Compared with the cone and 3D convex hull volumes, the crown volumes predicted by our profile models had significantly smaller errors. The results revealed that the crown profile can be well described by using UAV-LiDAR, providing a novel way to obtain crown profile information without destructive sampling and showing the potential of the use of UAV-LiDAR in future forestry investigations and monitoring.

## 1. Introduction

The crown profile of a tree is the maximum outer edge of the crown branches and the minimum boundary that encapsulates the whole crown and can characterize both the shape and size of the crown [1]. The shape and size of the crown affect tree physiological processes, such as photosynthesis, respiration and transpiration, due to the utilization of light and precipitation [2,3]. Additionally, the crown profile reflects a tree’s characteristics and growth (such as its species, age, and size) [1,4,5,6] and the tree’s response to the surrounding environment, such as competition and site conditions [5,7], and is thus closely related to species diversity and ecosystem stability [8].

A variety of models have been used to describe crown shape and size and predict crown width at any location within the crown. Such models were initially based on simple geometric shapes (e.g., ellipsoid or cone) [9,10]. However, simple geometric shapes lack flexibility, leading to inaccuracy. Therefore, numerous researchers have developed direct and indirect models to describe crown shape and size. In indirect methods, branch attributes (e.g., branch length and angle) and their trigonometric relationships are usually used to model the crown profile [11,12], whereas in direct methods, some easily measured tree attributes, such as tree height (TH), crown length (CL), and diameter at breast height (DBH), are used to predict the crown profile [13,14]. Compared with indirect models, direct models require fewer field measurements and have superior adaptability. Going beyond using a single model, some authors divided the crown into the upper crown (primarily sun branches) and the lower crown (primarily shade branches) based on the largest crown radius (LCR), and two equations were then used to describe the crown profile; the results using two equations to estimate the upper and lower crown shape were better than those obtained using a single equation to estimate the shape of the entire crown [3,15,16].

Traditionally, destructive sampling is carried out to obtain data for crown profile modelling, the attributes of branches are measured, and the crown radius is calculated with trigonometric methods [1]. Although manual measurement methods can be used to obtain accurate crown radius values at any position, they are extremely time consuming and labour intensive [17]. The development of modern measurement technology can improve efficiency and reduce both labour and material resources; for example, remote sensing (especially active remote sensing) techniques can be used instead of manual measurements to obtain variables for modelling crown profiles [18].

Light detection and ranging (LiDAR) is an active remote sensing technology that can penetrate the canopy and effectively capture ground points and provide data on vertical canopy structure and terrain [19,20]. It can be used to capture three-dimensional (3D) tree crown attribute data and has been successfully applied to estimate forest parameters at the stand level [21,22,23] and tree level [24,25,26]. A large number of effective individual tree crown delineation (ITCD) algorithms have emerged in recent decades [27,28,29,30,31,32]. These ITCD algorithms offer a basis for extracting relatively fine-scale tree metrics and can facilitate the extraction of the crown profile. Furthermore, the LiDAR platform is constantly evolving, including space-borne laser scanning (such as ICESat-2), airborne laser scanning (ALS) (such as Geiger-mode LiDAR), especially near-ground platforms (such as unmanned aerial vehicle (UAV) laser scanning, terrestrial laser scanning (TLS), and mobile laser scanning (MLS)) that further provide the possibility of simulating the canopy profile.

As a type of near-ground LiDAR platform, UAVs benefit from lower material and operational costs and better data measurement flexibility and repeatability than aircraft and satellite platforms [33]. UAVs reduce the difficulties associated with extracting fine-scale tree data and can generate data with point densities of 100–300 points per square meter, or even up to 1000 points per square meter, representing a significant increase over the data provided by airborne laser scanning (ALS) [34], and they compensate for the limited scanning area associated with TLS [6,35]. Several researchers have managed to integrate LiDAR sensors with UAV platforms and further improve the accuracy of individual tree crown extraction [36,37,38]. On the basis of these studies, Wallace et al. [39] assessed the feasibility of UAV-based LiDAR using a set of descriptive statistics generated from LiDAR data and demonstrated the feasibility of the TerraLuma UAV-borne LiDAR. Wallace et al. [40] subsequently compared several individual tree detection and delineation algorithms with high-density UAV-LiDAR data, and the best-performing method correctly detected 98% of the individual trees in a four-year-old Eucalyptus globulus plantation. Jaakkola et al. [41] used a mini-UAV laser scanning method to automate tree-level field measurements, and a detection rate of 100% was achieved for isolated and dominant trees. However, most forestry applications based on UAV-LiDAR concentrate more on crown delineation and the extraction of individual tree metrics, such as TH and crown diameter [42,43], and they lack the simulation of species-specific crown profiles.

In summary, although UAV-LiDAR possesses unique advantages in terms of the collection of fine-scale measurements for forestry analysis, its potential for extracting tree crown information needs further investigation. *Larix olgensis* Henry is one of the most important coniferous and afforestation tree species in Heilongjiang Province, and the study of its crown profile is of great significance for the estimation of biomass and volume. Thus, the present study aimed to evaluate the feasibility of using a low-cost UAV-borne LiDAR system for crown profile modelling of *Larix olgensis*. The specific objectives were to (1) isolate individual trees from UAV-LiDAR data using a region-based hierarchical cross-section analysis (RHCSA) algorithm and estimate crown metrics (such as the LCR and CL) based on crown point clouds; (2) generate the most representative crown profile points from individual tree crowns using different crown-width percentiles (90th, 95th, and 99th) rather than through traditional destructive sampling; and (3) derive four crown profile models (parabola, Mitscherlich, power, and modified beta equations) for *Larix olgensis* at Maoershan Forest Farm from UAV-LiDAR data and compare their performance.

## 2. Materials and Methods

### 2.1. Study Area

**Figure 1:**
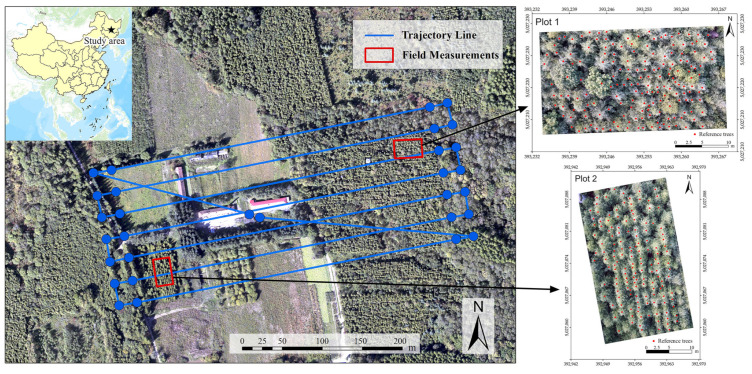
Location of the study area and schematic diagram of the unmanned aerial vehicle (UAV) route and reference data distribution.

The study area is at Maoershan Forest Farm, Shangzhi, Heilongjiang Province, Northeast China (Figure 1), ranging from 127°18′0″ to 127°41′6″ E and 45°2′20″ to 45°18′16″ N. The slope ranges from 5° to 25°, the terrain is high in the south and low in the north, and the average altitude is approximately 400 m. The study site is a typical natural secondary forest in Northeast China surrounded by various broadleaved trees, such as *Betula platyphylla*, *Quercus mongolica,* and *Populus davidiana*, and some coniferous plantations, such as those of *Larix olgensis*, *Pinus sylvestris*, and *Pinus koraiensis*. An 18-year-old *Larix olgensis* plantation located at the No. 3 ecological station of Northeast Forestry University was used for data collection.

### 2.2. Data Collection

#### 2.2.1. UAV-Borne LiDAR Data

The UAV-borne LiDAR system used in this study was a low-cost 8-rotor UAV platform-based LiDAR system known as Li-Air (GreenValley Technology Co., Ltd., Beijing, China). It is composed of a Velodyne Puck VLP-16 laser scanner, a Novatel inertial measurement unit (IMU; SPAN-MEMS-IMU-IGM-S1), two global positioning system (GPS) antennae, a dual Novatel frequency GPS receiver, a micro-computer (called Li-Air One), and a Sony QX1 camera. As the dominant part of the system, the Velodyne Puck VLP-16 is the smallest laser scanner, supporting 16 channels at ~300,000 points/s, with a 360° horizontal field of view and a 30° vertical field of view, ±15° up and down. The maximum measuring range is 100 m, and the accuracy of the range measurements is ±3 cm. The footprint size was 18 cm in diameter, and the beam divergence was 3 mrad. The vertical and horizontal/azimuth angular resolution is 2.0° and 0.1°–0.4°, respectively. This system carries two 22,000 mAh batteries, which could support an ~20 min flight [36]. 

UAV-borne LiDAR data were captured on 4 July 2017. Flights took place at an altitude of 40 m above the ground, with a flight line spacing of 25 m and a flying speed of 3.6 m/s. The final point density was ~370 pt./m^2^ on average. During the data collection process, LiAcquire software (GreenValley Technology Co., Ltd., Beijing, China), which was developed by Guo et al. [36], was used to control the UAV system, monitor the real-time UAV flight parameters, and display the real-time acquired LiDAR data. To improve the georeferencing accuracy, Novatel Inertial Explorer software was used to generate flight trajectories and compute LiDAR point cloud coordinates with the IMU and GPS data. The simultaneous kinematic method developed by [44] was used to register the point clouds among the overlap strips, and the horizontal and vertical misalignment was less than 10 cm and 5 cm, respectively. Additionally, high-resolution images were simultaneously captured during the flights and used for visual interpretation.

#### 2.2.2. Reference Data

Field survey data were acquired simultaneously with the UAV data in 2017. Two experimental plots were established in the LiDAR data collection area, representing two stand densities (the initial planting densities were 1 × 1.5 m and 2 × 1.5 m). A total of 349 trees in two plots were measured to obtain reference data to match with the LiDAR data. The DBH, TH, and crown radius (CR) in four directions were measured for each tree. In addition, the absolute coordinates of the four corners of the plots and the relative coordinates of the trees were recorded to accurately match with the LiDAR data. At the same time, the high-resolution images from the UAV were used to correct the positions of the reference trees (Figure 1). In total, 203 locations of trees in Plot 1 were collected, including those of 13 dead trees, 13 other trees (e.g., *Fraxinus mandshurica*, *Ulmus pumila,* and *Betula platyphylla*) and one tree with a DBH value <5 cm, while 146 locations of trees in Plot 2 were collected. Summary statistics for all *Larix olgensis* trees with DBH values ≥5 cm in the two plots are listed in Table 1.

### 2.3. Methods

**Figure 2:**
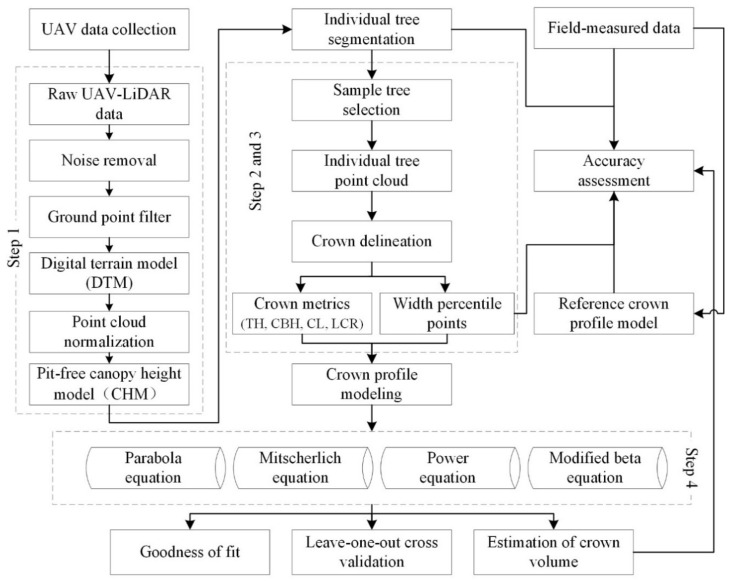
An overview of the workflow for modelling crown profiles using unmanned aerial vehicle (UAV) laser scanning data. TH: tree height; CBH: crown base height; CL: crown length; LCR: largest crown radius.

An overview of the workflow for modelling crown profiles using UAV laser scanning data is shown in Figure 2. First, the raw UAV-LiDAR data were preprocessed, and a canopy height model (CHM) was generated. Second, individual trees were detected, and the crowns were delineated from the CHM using a RHCSA algorithm. Third, the crown variables were extracted from each crown for crown profile modelling. Finally, four equations (parabola, Mitscherlich, power, and modified beta equations) were used to model the crown profile of *Larix olgensis*, and the accuracy of the models and the crown volumes derived from the models was evaluated. These steps are further elaborated in Section 2.3.1, Section 2.3.2, Section 2.3.3, Section 2.3.4 and Section 2.3.5. All methods were coded in MATLAB R2019b.

#### 2.3.1. UAV-LiDAR Data Preprocessing

The raw UAV-LiDAR data have a number of noise points, which can be divided into three categories: air points, low points, and isolated points. Air points and low points were removed manually, and isolated points were determined by the number of points inside the search neighborhood for a given search radius (5 m). Then, ground and nonground points were separated using the progressive triangulated irregular network (TIN) densification method developed by Axelsson [45]. The ground points were then interpolated into a digital terrain model (DTM) using kriging interpolation [46]. The normalized height of the point clouds was obtained by subtracting the DTM value from the elevation of all points [29]. Subsequently, graph-based progressive morphological filtering (GPMF) was applied to generate pit-free CHMs for subsequent individual tree segmentation [47]. The CHM generated with the GPMF method has smoother canopy surfaces with fewer data pits than the CHM directly interpolated with the first returns while preserving the edges, shape, and structure of the canopy gaps and crowns.

#### 2.3.2. Individual Tree Segmentation and Sample Tree Selection

**Figure 3:**
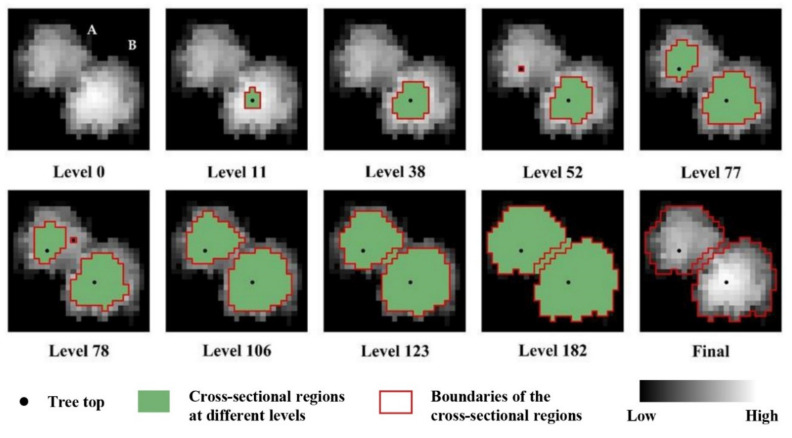
Schematic diagram of the region-based hierarchical cross-section analysis (RHCSA) algorithm based on a canopy height model (CHM) containing tree A and tree B.

To explore the ability to develop crown profile models using UAV-borne LiDAR, individual tree segmentation should be first carried out to obtain individual crown point clouds. In this study, a RHCSA algorithm was introduced to automatically detect individual trees. This algorithm considers the CHM to be a mountain-like topographic surface and utilizes horizontal relationships among crowns in the vertical direction to detect individual trees. Specifically, the RHCSA algorithm slices the CHM with a series of equidistant horizontal planes from top to bottom. Each cut represents a level, and the CHM was resolved into horizontal crown regions at different levels in vertical space (see Figure 3). The highest tree (tree B) produced a cross-section earlier than the shortest tree (tree A) (Level 11 in Figure 3). The first emerged region that did not contain any cross-sectional region at the previous level was defined as a marker (Levels 11, 52, and 78, in Figure 3), and the cross-section gradually increased in diameter with increasing level. In general, the shape of the cross-section region is similar to a circle; when a cross-section contains more than one marker, invalid markers (often produced by branches) were eliminated by its circularity (Level 78 and 106, in Figure 3). In contrast, the cross-sections produced by multiple contacted trees often appear irregular in shape, and these cross-section regions were separated by marker-controlled watershed algorithm (Level 123 and 182, in Figure 3). After segmentation, a pixel-based binary morphology opening operation was applied to refine the segment boundaries and remove the irregular segment objects. In the RHCSA algorithm, each level cut represents one iteration. Individual tree crowns and treetops are completely extracted until all iterations end (until level cutting reaches the final layer). The details of the RHCSA algorithm can be found in Zhao et al. [30]. 3D tree point clouds were extracted for each tree from the CHM-based crown delineation region in vertical space to ensure the completeness of the tree crown data and reduce the loss of detail.

After segmentation, trees that met the following requirements for modelling the crown profile were selected. First, the detected trees that were 1:1 matched to the field measurements were selected. The matching rule between detected and reference trees was developed by Reitberger et al. [48]. The distance to the reference tree is less than 60% of the average tree distance within the plot, and the height difference between the detected TH and reference TH is less than 15% of the greatest TH in the plot. If a reference tree is assigned to more than one detected tree, the tree closest to the reference tree is deemed to be a 1:1 matched tree. Then, dead trees, trees with a DBH value <5 cm and other tree species (e.g., *Fraxinus mandshurica*, *Ulmus pumila*, and *Betula platyphylla* in this study) were removed. Since accurate shape simulation can be carried out only with complete crowns, the most important and necessary step is to check the integrity of the individual tree crowns. Therefore, we conducted rigorous crown selection through visual inspection.

#### 2.3.3. Estimation of Model Variables

##### Estimation of Crown Metrics

**Figure 4:**
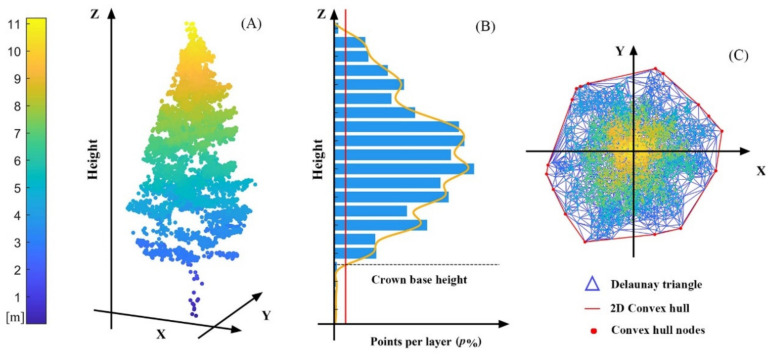
(**A**) Individual tree point clouds, (**B**) determination of the crown base height, and (**C**) the vertical projection of the crown for crown width estimation.

The complete point cloud of a tree is the nonground point cluster of stem points and crown points (Figure 4A). Characterization of the crown profile is predicated on identifying the crown base, which is defined as the height to the first living branch in traditional measures. Herein, the crown base height (CBH) was defined as the height where the number of tree point changed abruptly in the vertical direction. Specifically, all tree points were divided into 0.5 m bins from bottom to top, and the percentage of the number of points ($n_{i}$) per layer in relation to the total number of points per tree ($n_{\mathrm{tree}}$) formed the vector $N_{p}=\left\{{100\;\times \;n}_{i}{/N}_{\mathrm{tree}}\right\}$. Then, $N_{p}$ was smoothed with a 3 × 1 Gaussian filter, and the CBH was defined as the height that corresponds to p% of the total number of tree points (Figure 4B). To enhance the adaptability of our data, p% was ultimately set to 1% according to the highest accuracy (the smallest root mean square error (RMSE)) of verification between the detected value and the measured value.

The CBH was used to extract the crown from individual tree points, and a series of crown characteristic parameters were used as future estimates. The highest point within each tree crown was regarded as the tree top, and its x,y coordinate and z value represented the tree location and the TH, respectively. The CL, which represents the crown size in the vertical direction, was calculated by subtracting the CBH from the TH. Additionally, the vertical projection of the crown was used to estimate the LCR by constructing a two-dimensional (2D) convex hull algorithm (Figure 4C). The average value of the distance from the convex hull nodes to the vertex of the tree crown was defined as LCR. The TH, CL, and LCR were used as further parameterized variables to reflect the variation in individual tree size in the crown profile modelling procedure.

##### Width Percentile Generation

To eliminate asymmetrical branches due to competition between trees, the crown points of each tree were converted from 3D space to 2D space [6]. The vertical direction of the tree top was regarded as the central axis of the crown. The horizontal Euclidean distance between each crown point and the central axis was then calculated to generate a 2D distribution of the crown returns. In the new XY space, the x-axis measured horizontal distance from the central axis and the y-axis measured height above ground.

**Figure 5:**
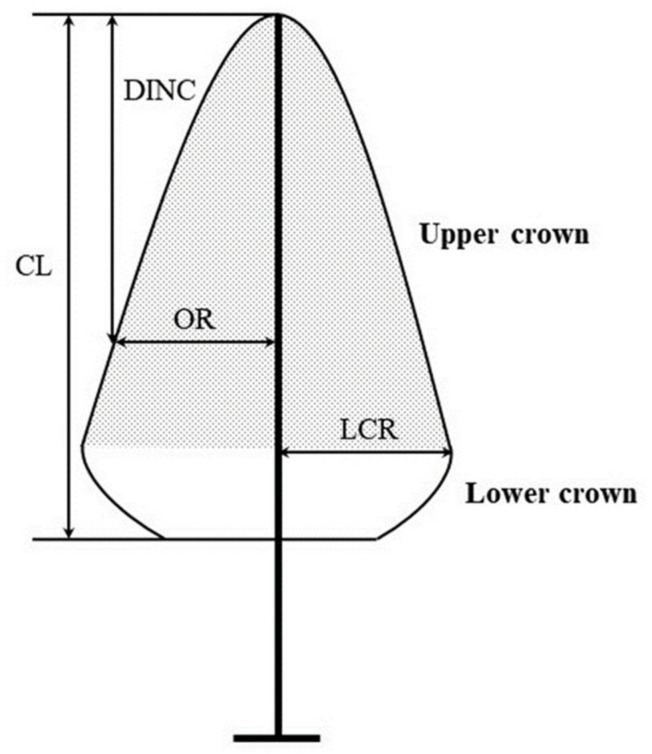
A schematic representation of the crown variables used for crown profile modelling. CL: crown length; OR: outer crown radius, DINC: depth into the crown; LCR: largest crown radius.

A 0.5 m height bin was used to divide each 2D distribution of crown returns from the tree top to the crown base, and the cumulative width percentiles were calculated within each bin [6]. To adequately describe the outer limit of each crown and to remove outliers, the 90th, 95th, and 99th percentiles were used to generate the crown profiles. Then, the width percentile points were vertically rescaled to between 0 and 1 to facilitate comparisons among trees of different CLs. Specifically, the vertical distance from each width percentile point to the tree top (the depth into the crown, DINC) was calculated, and this value was converted into relative depth into the crown (RDINC) by dividing it by the CL. Taking RDINC as the independent variable, a crown profile model was used to describe the outer crown radius (OR) at different crown positions, and the model variables are shown in Figure 5.

#### 2.3.4. Crown Profile Modelling

Four basic equations (parabola, Mitscherlich, power, and modified beta equations) derived from the existing crown profile model were used to fit the aggregated width percentile points in this study. To make the curve reasonable in describing the outer crown profile of conifer trees, the OR should be restricted to 0 when the RDINC is 0 [1]. Hence, the intercept term of the equations was removed. Because the parameter estimates of the models varied across individual trees, crown metrics were introduced into the basic model by analyzing the relationship between the parameters and evaluated tree metrics (TH, CL, and LCR). The LCR, as the variable with the highest correlation with the other parameters, was introduced into the equation last, and the specific forms of the four reparameterized models are as follows.

The parabola equation is the equation most widely used to describe the outer crown profile due to its flexibility [5,8,49], and its reparameterized form is shown below as Equation (1).

(1) $${\mathrm{OR}\;=\;(a}_{1}{\;+\;a}_{2}{\mathrm{LCR})\mathrm{RDINC}\;+\;\mathrm{bRDINC}}^{2}$$

where *OR* is the outer crown radius; *RDINC* is the relative depth into the crown; *LCR* is the largest crown radius; and $a_{1}$, $a_{2}$, and b are parameters to be estimated.

The Mitscherlich equation is suitable for describing tree growth characterized by faster growth at the beginning, which is similar to the growth of the crown at the top; hence, it was used to simulate the crown branches [50]. Here, we used this equation and reparameterized it as in Equation (2).

(2) $${\mathrm{OR}\;=\;a(1\;-\;e}^{{-(b}_{1}{\;+\;b}_{2}\mathrm{LCR})\mathrm{RDINC}})$$

where *a*, $b_{1}$, and $b_{2}$ are parameters to be estimated.

The power function and its transformation form are often used to model the crown profile [5,49]. The reparameterized model is given in Equation (3).

(3) $$\mathrm{OR}\;=\;\left(a_{1}{\;+\;a}_{2}\mathrm{LCR}\right){\mathrm{RDINC}}^{\left(b_{1}{\;+\;b}_{2}\mathrm{LCR}\right)}$$

where $a_{1}$, $a_{2}$, $b_{1}$, and ${\;b}_{2}$ are parameters to be estimated.

A 3-parameter beta function developed by Ferrarese et al. [6] was also used in this study and is given in Equation (4).

(4) $$\mathrm{OR}\;=\;\left(c_{1\;}{+\;c}_{2}\mathrm{LCR}\right)\frac{{\left(1\;-\;\mathrm{RDINC}\right)}^{a-1}{\mathrm{RDINC}}^{\left(b_{1}{\;+\;b}_{2}\mathrm{LCR}\right)-1}}{\beta \left(a,\;\left(b_{1}{\;+\;b}_{2}\mathrm{LCR}\right)\right)}$$

where a, $b_{1}$, ${\;b}_{2}$, $c_{1\;}$, and $c_{2}$ are parameters to be estimated.

All the above extracted crown profile points were employed to fit these four models with nonlinear least square fitting. These four models were also used to simulate the upper crown (also called the light crown), which is the portion of the crown above the point where the LCR occurs.

#### 2.3.5. Accuracy Assessment

In this study, the accuracy assessment included three parts: individual tree matching between the detected and reference trees, comparison between UAV-LiDAR crown profile points and reference values, the validation of the four crown profile models presented above, and the evaluation of crown volume prediction.

The widely used summary metric of detection accuracy (DA) was used in this study to quantify the accuracy of tree detection [43]. DA is calculated as the ratio of the number of 1:1 detected trees to the number of all reference trees.

The assessment of crown profile point accuracy is performed to evaluate the correspondence between the crown radius and the profile points. Since it is difficult to measure the crown radius at all positions within the corresponding sample tree crown, we used the *Larix olgensis* crown profile model developed by Gao [51] (Equation (5)) to calculate the reference data and verify the three width percentile points (90th, 95th, and 99th) from the UAV-LiDAR data.

(5) $${OR\;=\;(a}_{1}{\mathrm{DBH}}^{a_{2}}){\left[\frac{{1\;-\;(1\;-\;\mathrm{RDINC})}^{0.05}}{1\;-\;{\left(a_{3}{\mathrm{CH}}^{a_{4}}\right)}^{0.5}}\right]}^{a_{5}{(1\;-\mathrm{RDINC})\;+\;a}_{6}\left(\exp(1/\mathrm{HD})(1\;-\mathrm{RDINC})\right)}$$

where *DBH*, *CH* (ratio of *CL* to *TH*), and *HD* (ratio of *TH* to *DBH*) are field-measured values, and the procedure for the estimation of parameters $a_{1}$–$a_{6}$ can be found in Gao [51]. The Pearson correlation coefficient (*ρ*), RMSE, relative RMSE (RMSE%), Bias, and relative Bias (Bias%) [43] were used to evaluate the accuracy and error of our estimated and reference values (Equations (6)–(9)).

(6) $$\mathrm{RMSE}\;=\;\sqrt{\frac{\sum_{i=1}^{n}{{(y}_{L}\;-{\;y}_{G})}^{2}}{n}}$$

(7) $$\mathrm{RMSE}\%\;=\;100\;\times \;\frac{\mathrm{RMSE}}{\bar{y_{G}}}$$

(8) $$\mathrm{Bias}\;=\;\frac{\sum_{i=1}^{n}{(y}_{L}\;\;{-\;y}_{G})}{n}$$

(9) $$\mathrm{Bias}\%\;=\;100\;\times \;\frac{\mathrm{Bias}}{\bar{y_{G}}}$$

where *n* is the number of crown profile points, $y_{L}$ is the estimated *OR* from UAV-LiDAR data, and $y_{G}$ is the predicted *OR* from Gao [51]’s model.

R^2^ and the RMSE were used to compare the goodness of fit of the four models. Leave-one-out cross validation was used to verify the predictive effect of each model [52]. Three statistical criteria were calculated: mean prediction error (MPE), mean absolute error (MAE), and mean relative absolute error (MAE%) (Equations (10)–(12)).

(10) $$\mathrm{MPE}\;=\;\frac{\sum_{i=1}^{n}\left(y_{i}\;{-\hat{y}}_{i}\right)}{n}$$

(11) $$\mathrm{MAE}\;=\;\frac{\sum_{i=1}^{n}\left|y_{i}\;{-\hat{y}}_{i}\right|}{n}$$

(12) $$\mathrm{MAE}\%\;=\;\frac{\sum_{i=1}^{n}\left(\frac{\left|y_{i}\;{-\hat{y}}_{i}\right|}{y_{i}}\right)\;\times \;100}{n}$$

where *n* is the number of samples, $y_{i}$ is the observed value of the *i*th sample, and ${\hat{y}}_{i}$ is the predicted value of the model.

In order to further evaluate the prediction accuracy of these four models, individual tree crown volumes were derived from the rotation of each profile. Since crown volume is hard to measure through field survey, the crown volumes predicted by Gao [51] were calculated as the reference volumes. As a comparison, a simple geometry cone was used to represent tree crown shape [6], and the radius of the cone was the measured LCR and the height was the measured CL. Another commonly used LiDAR volume estimation method, 3D convex hull [53], was also used for comparison. Crown volumes were assessed in terms of the absolute error produced by each model. Paired T-test was used to further evaluate the differences between every two volumes.

## 3. Results

### 3.1. Individual Tree Segmentation and Sample Tree Selection

The results of individual tree segmentation with the RHCSA algorithm are shown in Table 2. A total of 270 detected trees were successfully matched with the measured trees, and the DA was 77.4%. The DA of Plot 2 was higher than that of Plot 1, which is due to the easier separation of trees in Plot 2 with lower stand density. On the other hand, a few deciduous trees with irregular branches tended to be over-segmented, which may have decreased the DA of Plot 1. Then, nine other trees (including five *Fraxinus mandshurica* trees and four *Betula platyphylla* trees) were removed from all 1:1 matched trees. A few incomplete crowns were also removed. As a result, 243 out of 270 (90%) matched trees were selected for the next experiment after the inspection of tree crown integrity.

### 3.2. Comparison of Width Percentile Points

After the selection of sample trees, the points comprising the 90th, 95th, and 99th width percentiles for each tree were generated. As a result, 2392 points were generated for each width percentile. The width percentile points of each tree have two attributes: one is the position of the crown (RDINC) after rescaling, and the other is the corresponding OR. Reference data calculated according to Equation (5) were used to verify the OR estimated based on the UAV-LiDAR data, and the results show a stronger correlation between the measured value and the estimated value (*ρ* of 0.864) and that there were smaller RMSE (0.3354 m) and RMSE% (24.49%) values for the 95th width percentile than for the 90th and 99th percentiles (Table 3). In terms of Bias and Bias%, we found that the 99th width percentile overestimated the crown radius, while the 95th and 90th width percentiles underestimated the crown radius. When the selected percentile is large, a more extended crown is clearly observed, which may lead to the overestimation of the crown radius. Therefore, we suggest that the 95th width percentile is the most suitable for describing the outer profile of tree crowns. Even when the differences among the three width percentiles were small, the 95th percentile points were used in the crown profile fitting procedure.

### 3.3. Model Fitting and Validation Results

The model parameters and the goodness of fit of the curves generated from the 95th width percentile points are given in Table 4. It is noteworthy that all parameters were significant (*p* < 0.05), and the results of the goodness-of-fit statistics for the four models illustrate that all crown profile models had a high goodness of fit (R^2^ > 0.82). The modified beta equation (Equation (4)) showed the best performance, with an R^2^ of 0.859 and a RMSE value of 0.2433 m. The parabola equation showed suboptimal performance, with an R^2^ of 0.857 and a RMSE value of 0.2448 m. For the upper crown, the results showed that it had better fitting results than the entire crown regardless of equation type. The modified beta equation still showed the best performance and explained nearly 90% of the observed variability, with an RMSE value of 0.2240 m. The power and parabola equations showed slightly lower accuracy than the modified beta equation. The Mitscherlich equation showed the worst performance in fitting the crown profiles from the UAV-LiDAR data. In terms of the number of parameters in the model, the parabola equation has the advantages of few parameters and high accuracy.

Table 5 presents the leave-one-out cross validation results for the four crown profile models. In terms of the MPE, the entire crown radius was slightly underestimated by the power equation and slightly overestimated by the other equations. The modified beta equation had the smallest MAE and MAE%, and the Mitscherlich equation had the largest MPE, MAE and MAE% for the entire and upper crown. 

### 3.4. Evaluation of Crown Volume from Profile Models

**Figure 6:**
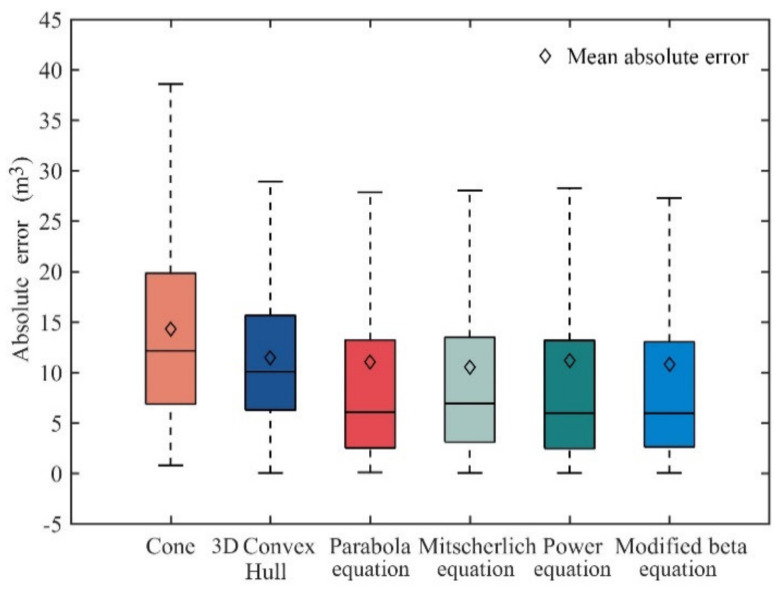
Comparison of crown volume prediction for the six models by absolute error.

The absolute errors of the predicted volumes derived from six models (cone, 3D convex hull, parabola, Mitscherlich, power, and modified beta model) were shown in Figure 6. The results showed that the cone volume has the biggest error, whereas the modified beta volumes have the smallest error among all volumes. The four volumes obtained from the profile model we developed have relatively small errors compared with the cone and 3D convex hull volumes. In terms of mean absolute errors, except for cone, the five volumes were relatively small. For volume prediction results, there was no significant difference among the four volumes predicted by profile models (*p* > 0.05), but significant difference between predicted volumes of each profile model and volumes of the cone or 3D convex hull (*p* < 0.05). 

## 4. Discussion

In the present study, we applied low-cost eight-rotor UAV platform-based LiDAR, which can quickly provide dense returns for ground objects to obtain the external crown shape of *Larix olgensis*. The results demonstrate that UAV-LiDAR can be used to model crown profiles, especially for the upper crown. Here, we will further discuss the feasibility of using UAV-LiDAR for modelling crown profiles and factors that affect such modelling as well as provide suggestions for future work.

### 4.1. Feasibility of Modelling Crown Profiles Using UAV-LiDAR

Crown profile extraction benefits greatly from the flexibility of UAV-LiDAR. First, UAV-LiDAR produces higher-density points than ALS by flying at a relatively low altitude and slow speed [33]. Hence, it can provide more detailed characteristics of forest canopy structures in a small region than ALS. Second, UAV-borne systems have higher measurement precision than satellite and airborne systems. A Velodyne Puck VLP-16 sensor was used in this study as the scanner, and the accuracy of the measured distance was ±3 cm [36]. In contrast, typical satellite and airborne laser sensors have a larger footprint, which causes greater errors in the positioning of the laser returns [41]. Third, UAVs are flexible and efficient in terms of data acquisition. For plot level scanning, a systematic multi-scan location approach and subsequent co-registration are necessary and siting retro-reflective targets can also be time-consuming when using TLS [54], whereas UAVs can plan routes intelligently and complete automatic acquisition within a few minutes. For similar resolution levels and in particular same goals, e.g., obtaining the crown profiles, UAV can achieve the same effect as TLS under the premise of high efficiency. Of course TLS will take more time but this is a trade-off between time and accuracy and detail. Furthermore, while UAV may be well suited for approximating crown profiles and TLS might be “too much” for that purpose, in other tree metrics (e.g., DBH) TLS can be much better than UAV based scanning. 

In the initial step of modelling the crown profile in this study, individual crowns were delineated with 77.4% overall accuracy (81.5% and 74.4% in Plots 1 and 2, respectively). It is noteworthy that our target trees were planted at high density (the stand densities of Plots 1 and 2 were 3167 n/ha and 2433 n/ha, respectively). Previous studies have shown that the detection rate of trees decreases with increasing tree density [55], and they pointed out that when the number of trees per hectare is greater than 1500, the detection rate decreases below 0.5 when ALS data are used. Wu et al. [56] used the same UAV-LiDAR system employed in this study to segment individual trees with four algorithms in three stem density plots, and the results showed that the accuracy of the segmentation was between 74% and 80% at the high stem density (713 n/ha). In view of the high stand density in this study area, the segmentation algorithm used in this study had a great effect on the UAV-LiDAR crown delineation. The accuracy of individual tree detection could even be improved in sparse forests.

**Figure 7:**
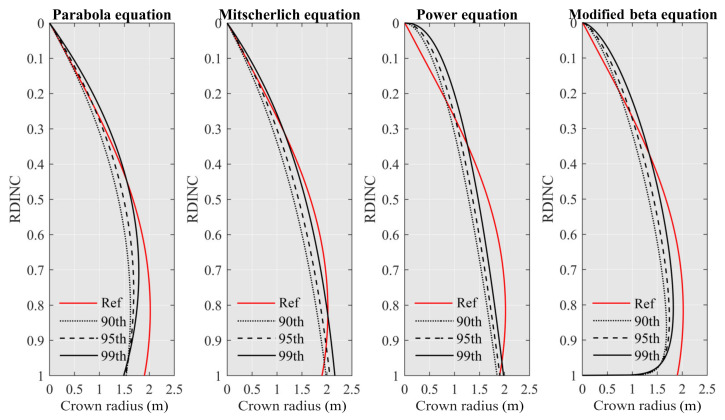
Four types of curves modelled on 90th, 95th, and 99th width percentile points; red curves represent the reference crown profile.

For the selection of modelling variables, profile points were generated from 2D crown points using width percentiles. The width percentile adequately describes the outer limit of each crown [35]. Compared with the 100th percentile, a position interior to the 100th percentile can compensate somewhat for horizontally asymmetrical crowns [6]. Herein, we compare the 90th, 95th, and 99th width percentiles in terms of crown profiles predicting (shown in Figure 7). For comparison, the crown profile generated by Gao [51]’s model (Equation (5)) was also overlaid onto the figure. To quantify the differences among the various curves, the RDINC of the tree was divided into 100 intervals, and the average RMSE of these intervals between the 90th and 95th percentiles and between the 99th and 95th percentiles were 0.0786 m and 0.1073 m, respectively. The differences among the three width percentiles were found to be small, and these three curves are similar to the reference curve (the smallest RMSE was 0.0885 m). This indicates that the width percentiles from UAV-LiDAR data are reliable for predicting crown profiles.

When the 95th percentile points were used for modelling and the LCR was introduced, the modified beta equation explained nearly 86% of the variability in the entire crown radius and nearly 88% of the upper crown radius variability, whereas the fitting accuracy of the basic model (without the LCR) was only 64% and 69% for the entire and upper crown, respectively. Many previous studies have used the LCR when developing crown profile models [10,57,58]. Crecente-Campo et al. [3] modified a simple polynomial equation and the model of Baldwin [2] by including the LCR in the model formulations, and the R^2^ was increased by 22.8% compared with that of the model without LCR values. Subsequently, Dong et al. [5] used this model and its modified form to generate the profile of Chinese fir, with fitting results of R^2^ values of 0.765–0.885 for the entire crown and 0.658–0.939 for the upper crown. Soto-Cervantes et al. [59] used six models containing the LCR to model the profile of *Pinus cooperi* Blanco, and the fitting results included R^2^ values of 0.467–0.918 for the entire crown and 0.854–0.984 for the upper crown. These results indicate that the four reparameterized models we used well represent the available information on the crown shape of *Larix olgensis* obtained from the UAV-LiDAR data.

Based on the results of the crown profile prediction, the modified beta equation achieved the best prediction accuracy, followed by the parabola model (Table 5). For the crown volume prediction, the modified beta and parabola equation still showed optimal performance (Figure 6). However, there was no significant difference in predicted volumes between the two models. The number of parameters in the parabola equation is two less than that of the modified beta equation, so it is flexible and convenient. The abovementioned results indicate that the model with three-parameters can accurately simulate the crown profile of *Larix olgensis* when using UAV-LiDAR data. In other studies on *Larix olgensis* crown profile, Gao et al. [7] compared four equations (segmented power equation, segmented polynomial equation, modified Weibull equation, and Kozak equation), and the number of parameters was 10, 8, 7, and 6, respectively. The Kozak equation with fewer parameters was selected as the best model when the fitting results are second only to 10-parameter segmented power equation. In this study, since the even age of trees leads to the small crown shape variation, the model with fewer parameters is suitable. If the data type is increased, the appropriate increase of model variables is meaningful for explaining the crown shape variation.

In traditional crown profile modelling, the sample tree was felled and tree metrics (such as DBH, TH, and CL) and branch attributes (such as branch length, branch chord length, branch angle, branch diameter, and depth into the crown) were measured. In this study, it is hard to measure the branch attribute of each tree. Nevertheless, we conducted a rigorous accuracy assessment through an established crown profile model of *Larix olgensis*, which was developed by Gao [51] (Equation (5)). In the proposed model, a total of 509 branches were measured from 49 felled sample trees. The samples with different ages, status, stand densities and slopes fully represent the distribution of *Larix olgensis* in Northeast China. After comparing five models (segmented parabola, segmented Mitscherlich, segmented power, modified Weibull, and Kozak equation), the Kozak equation was used as the best equation to model the crown profile. The tree variables (DBH, CH, and HD) were introduced into the model to describe the variation of crown shape. The profile model achieved high accuracy of fitting R^2^ of 0.83, and the crown volume estimated from the model achieved a high accuracy of 85%. These results indicate the reliability of the reference data.

This study provides a pipe-line from raw point cloud data to final crown profile predictions. Although we have only made profile prediction for *Larix olgensis*, the framework of this method can be transferred to other tree species, especially conifer species with similar shape (such as Korean pine, *Pinus sylvestris* var. *Mongolica*, etc.). Ferrarese et al. [6] have used the beta and Weibull equation to model the profile of different tree species (*Pseudotsuga menziesii*, *Pinus ponderosa*, and *Abies lasiocarpa*), the results indicate that there was no difference in accuracy between beta and Weibull curves for *A. lasiocarpa*, and both equations produced significantly small errors in all species. The four models we used have also been used by other researchers to model the crown profile of many other tree species (such as European beech, Chinese fir, Korean pine, *Pinus sylvestris* var. *Mongolia*). For broad-leaved species with complex crown shape, the current models may have limitations, the inverse third order polynomial equation can be a good choice [60].

### 4.2. Uncertainty in Modelling Crown Profiles Using UAV-LiDAR

Although UAV-LiDAR has great potential for modelling crown profiles, we also need to address the uncertainties in this process. By comparing the predicted value with the reference value (Figure 6), we found that the main source of the difference is the lower part of the crown (especially for the modified beta and parabola equations, which better fit the LiDAR percentile points, the underestimation becomes increasingly obvious with increasing RDINC), which directly reflects the lack of a description of the lower part of the crown by the UAV-LiDAR data. From the perspective of the data source, it is evident that UAV scanning above the canopy results in a decreasing return intensity from the top to the bottom of the canopy as well as the obstruction of the branches, thus reducing the number of points in the lower part of the canopy. Similarly, TLS struggles to identify points for the upper crown while scanning under the canopy. Other studies have also noted that occlusion was a major source of uncertainty and the difficulty of laser scanning and forest reconstruction in dense forests [54,61]. Therefore, we recommend using multi-return LiDAR sensors or full waveform recognition (with sizeable LiDAR footprints that potentially penetrate through the ground) to provide sufficient energy to better penetrate the canopy or combined UAV-LiDAR with ground-based LiDAR to generate a more complete canopy structure. 

In the process of data processing, individual tree segmentation and profile points generation are two key steps, which still exist some uncertainties. Although it has been proved that the RHCSA method obtained stable and high accuracy for different forest types, including coniferous forest, coniferous-broadleaves forest and deciduous forest, several limitations still exist. RHCSA considers CHM as mountain-like topographic surfaces, some flat crowns and suppressed trees without a dominant protrusion on CHM are difficult to detect and delineate [30]. In this study, the forest stand was an even-aged plantation, which has less suppressed trees and understory trees than the uneven-aged heterogeneous forests. The omission of a large number of suppressed trees and over segmentation of large trees may present great challenges and uncertainties for crown profile modelling. Hence, it is necessary to select the individual tree segmentation method according to the forest type. Additionally, it is difficult to distinguish the staggered parts of the canopy between adjacent trees with LiDAR, and thus the segmentation of individual trees will cause crown diameter loss, which is still a major challenge in individual tree segmentation [30]. Therefore, incomplete crown caused by staggering or occlusion needs to be removed by visual inspection. Although this work requires manual intervention, it is still acceptable compared with the workload of field measurement. For profile points generation, the uncertainty mainly comes from the selection of height bins. Since the detail of the profile description is determined by the size of the bin, the performance of the developed model will be sensitive to the bins. The smaller the bin, the more detailed the profile is, and the larger the bin, the more details are missing. In other studies, Ferrarese et al. [6] used the 0.25 m height increment bins to calculate width percentiles. However, due to the whorls of *Larix olgensis* from the sprouting branches between whorls [7], small bins will cause the crown profile to be affected by sprouting branches. Following Gao’s research [7], the branches were measured at 0.5 m intervals of the entire crown in detailed field measurement. Therefore, it is reasonable to select a height bin of at least 0.5 m. For other tree species, the height bins should be set according to the branch distribution characteristics and the requirement of level of details.

**Figure 8:**
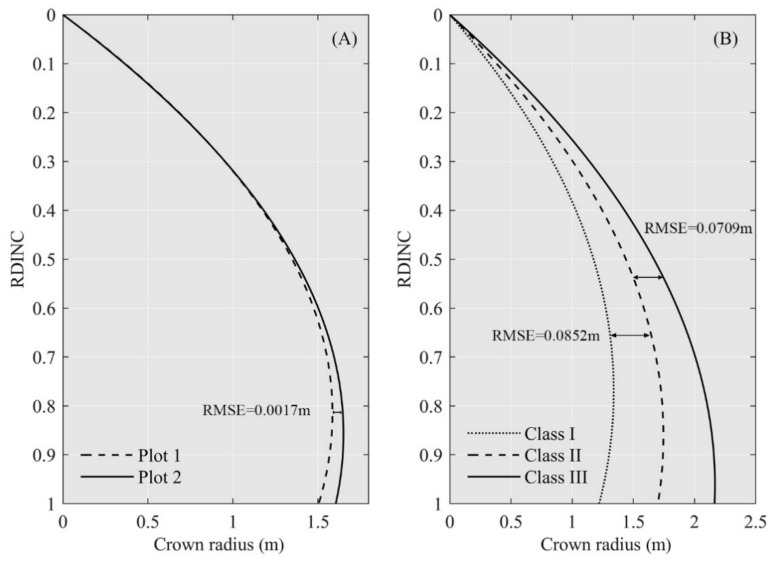
Curve fitting for (**A**) different stand densities and (**B**) different diameter classes using the parabola equation.

From a modelling perspective, the selection of sample trees should span a range of conditions and sizes. As noted previously, crown shape is affected by genetic characteristics and environmental variables, such as tree density, site productivity, and terrain [5,6]. In addition, crown shape also varies with the light conditions in different directions and competition with neighboring trees [7]. Although our study did not explicitly characterize the effects of multiple site conditions and competition with neighbors on crown shape, we further divided all sampled trees into two groups (Plot 1 and Plot 2) based on the stand density and three classes based on the DBH (Class I with DBH less than 10 cm; Class II with DBH between 10 cm and 13 cm; Class III with DBH greater than 13 cm) to analyze the effects of stand density and tree growth on the crown shape, respectively. The parabola equation was used for fitting, which has a similar accuracy to the beta equation and fewer parameters, leading to higher efficiency. The results of the fitting of the curves representing the data associated with the different stand densities (Figure 8A) show that the crown radii of individual trees at the lower stand density were larger than those at the higher stand density. This likely occurred because when the growth space is limited, the competition pressure experienced by the crown increases, which in turn limits the extension of the crown. In the future, more plots with different densities can be used to explore the relationship between the crown radius and tree density. The RDINC of the trees was divided into 100 intervals, and the RMSE of these intervals between Plot 1 and Plot 2 was 0.0017 m; this difference increased with an increase in the RDINC. In terms of the three diameter classes (Figure 8B), the RMSE between Class I and Class II was 0.0852 m, and that between Class III and Class II was 0.0709 m. The results indicate that the crown radius increased with DBH. Among trees of the same age, trees with a large DBH are more dominant, more competitive, and intercept more light, and they therefore grow better. It is clear from these results that the effect of tree growth on crown shape is consistent with the results of the study by Sun et al. [8].

Overall, modelling crown profiles using UAV-LiDAR showed great advantages, but there are also some noteworthy deficiencies. The main purpose of this study was to explore the feasibility of using UAV-LiDAR to model the crown profile. We found that the time commitment required for data acquisition, the efficiency of data processing and the accuracy of crown profile modelling are considerable compared with those associated with field-measured data or other LiDAR platforms. UAV-LiDAR may best be used to collect data from a large number of trees that are difficult to destructively sample. In addition, the data distribution is too centralized to represent the crown shape of various site conditions and age stages, and thus the crown profile models we developed can describe only the outer crown shape of trees in an 18-year-old *Larix olgensis* plantation. Additionally, UAV-LiDAR is somewhat labour intensive with regard to depicting the lower canopy structure, especially in the case of high tree densities, and individual tree segmentation is also one of the difficulties. With the emergence of a multi-return LiDAR sensor and the improvement of the branch and leaf separation algorithm, the lower crown could be better modelled.

## 5. Conclusions

In recent years, the accuracy of individual crown structures extracted with LiDAR has increased, and the emergence of UAV platforms has facilitated fine-scale crown shape descriptions. In this study, we explored the possibility of modelling the crown profile of *Larix olgensis* using UAV-based high-density LiDAR data, which are able to quickly characterize the crown extent in three dimensions without destructive sampling. By delineating individual tree crowns on the basis of UAV-LiDAR data and folding the 3D points representing each crown into 2D space, information about the extent of the entire crown was retained. Four equations (the parabola, Mitscherlich, power, and modified beta equations) were compared in terms of their performance in modelling the crown profile of *Larix olgensis* and showed good results.

Using high-point-density (~370 pt./m^2^) UAV-LiDAR data, we achieved a high accuracy of individual crown delineation (77.4%) in high-density (>2400 trees/ha) forest stands. The 95th width percentile is an adequate descriptor of the outer crown profile extracted from UAV-LiDAR point clouds when compared with the reference data (the Pearson correlation coefficient (*ρ*) was 0.864, RMSE = 0.3354 m), and little variation in the crown radius was detected when alternate width percentiles were used. When modelling the crown profile, the modified beta equation showed the best performance, explaining 85.9% of the observed variability for the entire crown and 87.8% of the variability for the upper crown. The parabola equation showed suboptimal performance, which is not significantly different from the modified beta equation in crown volume prediction and has fewer parameters. The volumes predicted by the four models produced significantly smaller errors than did cones or 3D convex hulls.

In summary, UAV-LiDAR displays excellent feasibility for extracting fine-scale tree crown shapes, especially for the upper crown. Occlusion among crowns and the lack of information below the crown remain two of the most confounding aspects of UAV-LiDAR for crown profile modelling. Future research should focus on supplementing information under the canopy by using multiple-return UAV-LiDAR or combined ground-based laser scanning and developing an accurate individual tree crown delineation algorithm to distinguish the branches from different crowns.

## Figures and Tables

**Table 1:** Summary statistics of *Larix olgensis* trees measured in the field.

	Variable	Mean	Minimum	Maximum	Standard Deviation
**Plot 1** (N = 176)	TH (m)	9.4	5.8	13.0	1.31
	DBH (cm)	9.7	5.1	16.7	2.76
	CR (m)	1.1	0.6	2.6	0.33
**Plot 2** (N = 146)	TH (m)	10.1	6.3	12.7	1.18
	DBH (cm)	10.6	5.2	16.5	2.59
	CR (m)	1.3	1.0	2.8	0.37

Note: TH is tree height, DBH is diameter at breast height, CR is crown radius.

**Table 2:** Results of individual tree segmentation and sample tree selection.

	Reference Trees	Detected Trees	1:1 Matched Trees	Detection Accuracy	Final Selected Trees
**Plot 1**	203	220	151	74.4%	129 (63.5%)
**Plot 2**	146	142	119	81.5%	114 (78.1%)
**Total**	349	362	270	77.4%	243 (69.6%)

**Table 3:** Results of the comparison of the LiDAR-estimated crown profile against field-measured model values.

	ρ	RMSE (m)	RMSE%	Bias (m)	Bias%
90th width percentile	0.860	0.3619	26.41	−0.1541	−11.25
95th width percentile	0.864	0.3354	24.49	−0.0830	−6.06
99th width percentile	0.854	0.3388	24.53	0.0112	0.81

Note: ρ is Pearson correlation coefficient, RMSE is root mean square error.

**Table 4:** Estimates of the parameters and goodness-of-fit statistics for the four crown profile models obtained from the 95th width percentile points.

		Entire Crown				Upper Crown			
	Parameter	Estimate	Standard Error	R^2^	RMSE (m)	Estimate	Standard Error	R^2^	RMSE (m)
Parabola equation	$a_{1}$	1.3512	0.0216	0.857	0.2448	1.4034	0.0244	0.873	0.2287
	$a_{2}$	1.8672	0.0460			−1.9490	0.0544		
	*b*	−2.2775	0.0487			1.6310	0.0498		
Mitscherlich equation	*a*	2.7232	0.0510	0.826	0.2699	3.1955	0.0845	0.853	0.2458
	$b_{1}$	−0.2816	0.0389			−0.0855	0.0297		
	$b_{2}$	1.0965	0.0494			0.8025	0.0435		
Power equation	$a_{1}$	0.1373	0.0425	0.847	0.2528	0.1522	0.0505	0.874	0.2273
	$a_{2}$	1.1891	0.0265			1.2768	0.0315		
	$b_{1}$	0.3847	0.0335			0.4408	0.0341		
	$b_{2}$	0.1202	0.0191			0.1278	0.0195		
Modified beta equation	*a*	1.0928	0.0070	0.859	0.2433	1.0435	0.0078	0.878	0.2240
	$b_{1}$	1.4175	0.0344			1.3783	0.0350		
	$b_{2}$	0.1787	0.0199			0.6785	0.0116		
	$c_{1}$	0.2125	0.0177			0.2246	0.0187		
	$c_{2}$	0.6590	0.0111			0.1966	0.0205		

**Table 5:** Validation results for the four crown profile models.

Model	Entire Crown			Upper Crown		
	MPE (m)	MAE (m)	MAE%	MPE (m)	MAE (m)	MAE%
Parabola equation	0.0149	0.1833	20.0521	0.0206	0.1755	20.2363
Mitscherlich equation	0.0295	0.2040	21.9694	0.0316	0.1883	21.4927
Power equation	−0.0012	0.1870	20.9585	0.0026	0.1715	19.8865
Modified beta equation	0.0034	0.1807	19.7303	0.0040	0.1699	19.7126

Note: MPE is mean prediction error, MAE is mean absolute error, MAE% is mean relative absolute error.
